# *Curcuma longa* normalized cimetidine‐induced pituitary‐testicular dysfunction: Relevance in nutraceutical therapy

**DOI:** 10.1002/ame2.12081

**Published:** 2019-09-02

**Authors:** Ngozi Joy Onwuemene, Christian Eseigbe Imafidon, Abiodun Oladele Ayoka

**Affiliations:** ^1^ Department of Physiological Sciences, Faculty of Basic Medical Sciences Obafemi Awolowo University Ile‐Ife Osun State Nigeria; ^2^ Department of Physiology, Faculty of Basic Medical and Health Sciences Bowen University Iwo Osun State Nigeria

**Keywords:** *Curcuma longa*, nutraceutics, pituitary, testis, Wistar rats

## Abstract

**Background:**

The increasing incidence of chemically induced infertility is both a social threat and a threat to the continuation of life itself. Treatment or management therapy is often expensive. This study investigated the effects of acetone extract of a local plant (*Curcuma longa*) in a Wistar rat model of cimetidine‐induced pituitary‐testicular dysfunction.

**Methods:**

Thirty‐five male Wistar rats were divided into 7 groups of 5 rats. After a phytochemical screening of an acetone extract of *C. Longa*, cimetidine and the extract at three doses, 200, 400 and 600 mg/kg, were orally co‐administered to the rats for 28 consecutive days. Comparisons were made (at *P* < 0.05) against a control (2 mL/kg distilled water), a standard treatment group (cimetidine + 50 mg/kg vitamin C), a toxic group (60 mg/kg cimetidine) and a group receiving extract alone.

**Results:**

Cimetidine administration was associated with deleterious alterations to sperm motility, sperm count and sperm viability, as well as derangements in the plasma levels of FSH, LH and testosterone (*P* < 0.05). Both brain and testicular GSH and TBARS levels were significantly altered following cimetidine administration, and distortions were seen in the pituitary and testicular histoarchitecture. These changes were significantly normalized by co‐administration of graded doses of the extract, with an associated improvement of both pituitary and testicular histology.

**Conclusion:**

Acetone extract of *C. Longa* normalized cimetidine‐induced pituitary‐testicular dysfunction in Wistar rats. This presents the extract as a potential nutraceutical choice against chemically induced reproductive toxicity.

## 1 INTRODUCTION

There is a gradual, but steady, increase in the incidence of chemically induced infertility globally.^1^, ^2^ Job demands and/or lifestyle may predispose an individual to health conditions that require long‐term regimens, which are often associated with deleterious effects on body organs, including those of reproduction.^3^ Damage to the reproductive organs threatens the continuation of life itself, and therefore the toxic effects of drugs and environmental toxicants on reproductive functions are becoming a major health concern worldwide.^2^, ^4^, ^5^

Acting as though they were a single unit, the hypothalamus, pituitary and gonads produce both local and systemic effects in the body by eliciting hormonal changes; hence they are referred to as the hypothalamic‐pituitary‐gonadal axis.^6^ This axis plays a crucial role in the control of development, reproduction, and aging.^6^, ^7^ Some drug regimens are known to cause fluctuations in this axis with consequent deleterious derangement of reproductive functions.^8^, ^9^, ^10^

Cimetidine is a globally prescribed potent drug that is commonly used for the treatment of gastric and duodenal ulcers.^11^, ^12^, ^13^ It is also readily available without prescription,^13^ thereby increasing its chances of being abused. Cimetidine is an H_2_‐receptor antagonist that blocks histamine action on H_2_‐receptors in the parietal cells of the stomach, consequently inhibiting acid production.^13^, ^14^, ^15^ Its administration has been found to be associated with reproductive toxicity and it is thus described as a reproductive toxicant.^8^, ^9^, ^13^ Some of the deleterious reproductive effects of cimetidine include distortion of testicular histoarchitecture with marked degeneration of the seminiferous tubules and maturation arrest of spermatogenic cells,^8^, ^9^, ^16^ decreases in sperm motility and count,^17^, ^18^ as well as impotence, with a resulting decrease in sexual drive and desire.^19^ Although the precise mechanism of cimetidine‐induced reproductive toxicity is uncertain, in males, cimetidine is known to target the hypothalamic‐pituitary‐testicular axis with associated structural changes in the histology of the testes.^16^ We therefore hypothesized that the normalization of reproductive function in subjects with cimetidine‐induced reproductive toxicity may be possible with intervention(s) that provide beneficial effects on both gastrointestinal and reproductive functions.

Even today, traditional medicine is a mainstay of primary health care in underdeveloped and developing countries.^20^, ^21^, ^22^, ^23^ Natural and herbal products from some plants are still very relevant in folk medicine for pharmaceutical formulations either as pure compounds or as extracts.^24^ Plant‐derived medicines are relatively cheap and readily available compared with their synthetic alternatives. They are also an important source of drug discovery and can inspire novel drug development.^21^, ^23^

*Curcuma longa* (Linn.), commonly called turmeric, is a perennial plant belonging to the Zingiberaceae family and is widely cultivated throughout the tropical parts of the world including India, China, Pakistan, Kenya, Ghana and Nigeria,^25^, ^26^, ^27^ making it readily accessible and cheap. It has a characteristic yellow colour that is conferred by its curcumin component.^24^ Some of the documented health benefits of this plant include anti‐inflammatory,^28^ antioxidant,^29^ anti‐carcinogenic,^30^ anti‐HIV,^31^ anti‐diabetic,^32^ lipid‐lowering,^33^ anti‐obesity,^34^ hepato‐protective,^33^ anti‐malarial^35^ and immunomodulating^36^ effects. The United States Food and Drug Administration (US FDA) classifies turmeric as a nutraceutic that is generally recognized as safe (GRAS).^37^, ^38^ According to a glossary produced by the American Diabetics Association, nutraceutics are substances that are considered as food or a part of food that offer medicinal health benefits, which include the prevention and treatment of diseases.^39^, ^40^

Despite the favorable ethnopharmacological properties of *C. Longa*, our literature survey revealed a dearth of information on the effects of its acetone extract on cimetidine‐induced pituitary‐testicular dysfunction. This study aimed to bridge this gap in our knowledge by assessing the nutraceutical effect of an extract of *C. Longa* in a Wistar rat model.

## 2 MATERIALS AND METHODS

### 2.1 Plants, drugs, chemicals, and biochemical kits

Fresh rhizomes of *C. Longa* were purchased from a commercial supplier at Sabo market in Ile‐Ife and certified by a Taxonomist at the Department of Botany, Obafemi Awolowo University (OAU), Ile‐Ife, where a voucher specimen (IFE‐17700) was deposited.

Cimetidine tablets were procured from Shandong Shenglu Pharmaceuticals, China. Vitamin C (analytical standard ascorbic acid) was from Nevada, USA. Acetone was of analytical grade. Standard laboratory hormone assay (biochemical) kits for testosterone, luteinizing hormone and follicle‐stimulating hormone for experimental rodents were supplied by Accu‐Bind Elisa (Monobind Inc).

### 2.2 Plant extraction process

The extraction process for obtaining an acetone extract of *C. Longa* rhizome (AECUL) followed the standard procedure described by Imafidon et al^41^ and Adekunle et al^42^ Fresh rhizomes of *C. Longa* were peeled and weighed. Thereafter, they were crushed in 80% acetone (1:2 w/v) with a Waring blender (Waring Commercial) for 5 minutes. The resulting mixture was homogenized using a polytron homogenizer for about 3 minutes and the homogenate was filtered under vacuum using a Buchner funnel and Whatman no. 2 filter paper (Whatman PLC). The filtrate was concentrated using a rotary evaporator under vacuum (40°C) and thereafter freeze‐dried in a lyophilizer (Ilshin Lab. Co. Ltd) at −40°C. The resulting acetone extract of *C. Longa* (AECUL) was weighed and kept in a desiccator until needed.

Acetone was used for the extraction process because it has been reported in the literature to extract high quantities of flavonoids and polyphenols from plant samples. These important phytochemicals have health‐boosting pharmacological activities.^41^, ^42^

### 2.3 Phytochemical screening of the extract

Phytochemical screening of the extract was carried out according to standard laboratory protocols. Alkaloids, flavonoids and tannins was detected by the method of Halilu et al,^43^ saponin was identified using the froth test as described by Benmedhdi et al,^44^ and phenolics were identified as described by Edeoga et al^45^ (Table 1).

**Table 1:** Phytochemical screening of acetone extract of *Curcuma longa* rhizome

Phytochemical constituents	Status
Flavonoids	+
Phenolics	+
Tannins	+
Alkaloids	+
Saponin	+

Note

**+ **= present.

### 2.4 Ethics statement

All experimental protocols were in strict compliance with the guidelines for animal research, as detailed in NIH *Guidelines for the Care and use of Laboratory Animals* 46 and were approved by the local institutional research committee.

### 2.5 Preparation of stock solutions of the extract

According to existing literature, the oral lethal dose of *C. Longa* is greater than 5000 mg/kg.^47^ Experimental dosage is usually taken to be less than or equal to 10% of the oral LD_50_.^41^, ^48^, ^49^ Therefore, the therapeutic doses of AECUL adopted for this study were 200, 400 and 600 mg/kg. In order to avoid deleterious biological effects due to fluid overload, stock solutions of the extract were prepared such that each 100 g rat received 0.02 mL (2 mL/kg).

The stock solutions were prepared by dissolving 2, 4 and 6 g of AECUL in 20 mL of distilled water, to provide the respective 200, 400 and 600 mg/kg doses in 0.02 mL.

### 2.6 Animal management

Thirty‐five male Wistar rats, weighing 150‐180 g, were used for this study. These were purchased from the Animal Holdings Unit of the College of Health Sciences, Obafemi Awolowo University, Ile‐Ife, Osun State, Nigeria, where the study was carried out. The rats were kept in standard plastic cages under a natural light and dark cycle and were allowed access to standard rodent pellets and water ad libitum.

### 2.7 Experimental design

The rats were divided into seven groups of five rats each and treated as follows. All groups received treatment for 28 consecutive days before they were euthanized. Group 1 received 2 mL/kg of distilled water; group 2 received oral cimetidine at 60 mg/kg; group 3 (standard treatment group) received co‐administration of cimetidine (60 mg/kg) and vitamin C (50 mg/kg); groups 4, 5 and 6 received oral co‐administration of cimetidine (60 mg/kg) and graded doses of AECUL at 200, 400 and 600 mg/kg, respectively; and group 7 received a single medium oral dose (400 mg/kg) of the extract (Table 2). After euthanasia, blood samples were collected by cardiac puncture into separate EDTA bottles and centrifuged at 4000 rpm using a cold centrifuge (Centrium Scientific, Model 8881) at −4°C. The plasma obtained was decanted into separate plain bottles. Thereafter, the caudal epididymis of each rat was excised and minced in 2 mL of normal saline and the resulting suspension was used for sperm characterization. The brain and testis of each rat were excised and weighed. The right testis and 1 g of the excised brain where transferred to a cooler for homogenate preparation, while the left testis and pituitary were fixed in 10% formal saline solution for histological examination using hematoxylin and eosin (H&E) staining.

**Table 2:** Experimental protocol and dose regimen

N = 35	Oral dose regimen	Days of administration
Group 1 (n = 5)	2 mL/kg distilled water	28^a^
Group 2 (n = 5)	Cimetidine (60 mg/kg)	28^a^
Group 3 (n = 5)	Cimetidine (60 mg/kg) + Vitamin C (50 mg/kg)	28^a^
Group 4 (n = 5)	Cimetidine (60 mg/kg) + 200 mg/kg AECUL	28^a^
Group 5 (n = 5)	Cimetidine (60 mg/kg) + 400 mg/kg AECUL	28^a^
Group 6 (n = 5)	Cimetidine (60 mg/kg) + 600 mg/kg AECUL	28^a^
Group 7 (n = 5)	400 mg/kg AECUL	28^a^

Abbreviations: AECUL, acetone extract of *Curcuma longa* rhizome; N, total number of rats used for the study; n, number of rats per group.

a Point at which rats were euthanized.

### 2.8 Determination of percentage weight change, relative brain weight, and relative testicular weight

Assessment of weekly weight change was carried out using a Hanson digital weighing scale (Hanson, China), while organ weights were determined using a Camry sensitive weighing balance (Camry, China). Thereafter, percentage weight change, relative brain weight and relative testicular weight were calculated using the formulae below 50:

$$\begin{array}{cc} \text{Percentage weight change}\;\left(\%\right)= & \frac{\text{Final body weight}-\text{Initial body weight}}{\text{Initial body weight}\;\left(g\right)}\\ \times 100\% \end{array}$$

$$\text{Relative brain weight}\;\left(\%\right)=\frac{\text{Whole brain}\;\left(g\right)}{\text{Final body weight}\;\left(g\right)}\times 100\%$$

$$\text{Relative testicular weight}\;\left(\%\right)=\frac{\text{Left testis}+\text{Right testis}\;\left(g\right)}{\text{Final body weight}\;\left(g\right)}\times 100\%$$

### 2.9 Sperm characterization

From the caudal epididymis of each rat, sperm fluid was squeezed onto a microscope slide. Sperm motility was assessed by counting the number of motile spermatozoa per unit area and was expressed as motility per unit area. Sperm counts were made with the aid of a hemocytometer and expressed as millions/ml of suspension. Sperm viability was determined by preparing a uniform smear of spermatozoa on the slides using eosin‐nigrosin stain according to the method of Bloom, 51 as described by Raji et al.^52^

### 2.10 Hormone assays

The concentrations of reproductive hormones (follicle stimulating hormone (FSH), luteinizing hormone (LH) and testosterone) were determined using standard laboratory kits involving the enzyme‐linked immunosorbent assay (ELISA) technique, according to the manufacturer's instructions.

### 2.11 Assessment of oxidative stress and lipid peroxidation indicators

A 10% homogenate of the tissue in phosphate buffer (100 mmol/L; 7.4 pH) was prepared using an electric homogenizer (S1601001). The homogenate was centrifuged at 3000 rpm for 20 minutes and the supernatant was decanted for assessment of indicators of oxidative stress. Reduced glutathione (GSH) was determined by the method of Beutler et al,^53^ while thiobarbituric acid reactive substance (TBARS) activity was determined by the method of Ohkawa et al.^54^

### 2.12 Histological examination

The pituitary and testis of the rats were dehydrated in graded alcohol and embedded in paraffin wax. Sections >4 µm thick were stained with hematoxylin‐eosin and photomicrographs were taken with a Leica DM750 Camera Micro at ×40 and ×100 magnifications for pituitary and testis, respectively.

### 2.13 Statistical analysis

The results were expressed as means ± standard error of mean using one‐way analysis of variance (ANOVA) followed by a Neuman‐Kuels post hoc test. Values at *P* < 0.05 were considered statistically significant. Data were analyzed using GraphPad Prism 5.03 statistical package (GraphPad Software Inc).

## 3 RESULTS

### 3.1 Effects of acetone extract of *Curcuma longa* (AECUL) on percentage weight change, relative brain weight and relative testicular weight of Wistar rats with cimetidine‐induced pituitary‐testicular dysfunction

Cimetidine administration (group 2) was not associated with significant changes in the percentage weight change (%), relative brain weight (%) or relative testicular weight (%) of the rats (*P* > 0.05). The same was true for the administration of AECUL alone (group 7) compared with the control (*P* > 0.05) (Table 3).

**Table 3:** Effects of acetone extract of *Curcuma longa* on percentage weight change, relative brain weight and relative testicular weight of Wistar rats with cimetidine‐induced pituitary‐testicular dysfunction

	Group 1	Group 2	Group 3	Group 4	Group 5	Group 6	Group 7
Percentage weight change (%)	24.50 ± 1.45	21.30 ± 1.38	23.45 ± 1.62	22.33 ± 1.29	24.20 ± 1.42	23.69 ± 1.51	23.77 ± 1.28
Relative brain weight (%)	1.48 ± 0.10	1.45 ± 0.15	1.47 ± 0.12	1.49 ± 0.11	1.46 ± 0.15	1.47 ± 0.13	1.23 ± 0.06
Relative testicular weight (%)	1.23 ± 0.04	1.21 ± 0.09	1.22 ± 0.07	1.24 ± 0.10	1.23 ± 0.10	1.22 ± 0.08	1.23 ± 0.06

Note

No significant difference was recorded at *P* < 0.05.

### 3.2 Effects of AECUL on sperm motility, sperm count and sperm viability of Wistar rats with cimetidine‐induced pituitary‐testicular dysfunction

Sperm motility was significantly lower (%) in group 2 compared with the standard treatment group 3 and the AECUL‐treated groups 4, 5 and 6 (*P* < 0.05). Sperm motility was also significantly reduced in group 3 compared with the control, and was insignificantly lower compared with the AECUL‐treated groups 4, 5 and 6. However, there was no significant difference in sperm motility in group 7 compared with the group 1 control (*P* > 0.05) (Table 4).

**Table 4:** Effects of acetone extract of *Curcuma longa* on sperm characterization of Wistar rats with cimetidine‐induced pituitary‐testicular dysfunction

	Group 1	Group 2	Group 3	Group 4	Group 5	Group 6	Group 7
Sperm motility (%)	85.90 ± 2.35	40.25 ± 2.50^a^	75.00 ± 2.85^a^,^b^	80.35 ± 2.40^b^	82.35 ± 2.20^b^	81.45 ± 2.60^b^	85.00 ± 2.00^b^
Sperm count (million/mL)	78.20 ± 2.55	40.20 ± 1.86^a^	71.30 ± 2.25^b^	70.60 ± 2.00^b^	74.80 ± 2.35^b^	75.50 ± 2.75^b^	75.40 ± 2.65^b^
Sperm viability (%)	80.55 ± 2.70	41.11 ± 2.80^a^	75.45 ± 2.65^b^	77.00 ± 2.55^b^	78.88 ± 2.20^b^	78.25 ± 2.80^b^	79.00 ± 2.40^b^

Note

Each value represents mean ± standard error of mean at *P* < 0.05.

a Significant difference compared with group 1.

b Significant difference compared with group 2.

Sperm count (millions/ml) and sperm viability (%) followed the same trend in our study. These parameters were significantly lower in group 2 compared with all other groups (*P* < 0.05). The standard treatment group 3 had an insignificantly lower sperm count and sperm viability compared with the AECUL‐treated groups 5 and 6. However, these parameters were not significantly different in group 7 compared with group 1 (Table 4).

### 3.3 Effects of AECUL on plasma levels of FSH, LH and testosterone of Wistar rats with cimetidine‐induced pituitary‐testicular dysfunction

**Figure 1:**
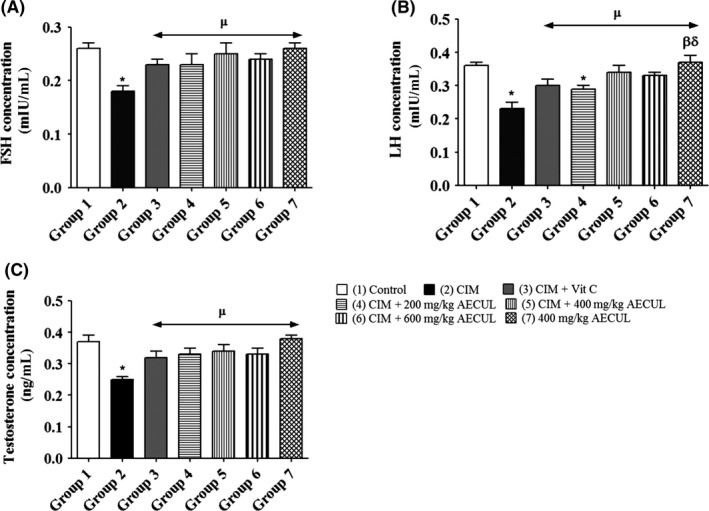
Effects of acetone extract of *Curcuma longa* on plasma levels of FSH, LH and testosterone in Wistar rats with cimetidine‐induced pituitary‐testicular injury. FSH, follicle stimulating hormone; LH, luteinizing hormone; CIM, cimetidine; Vit C, vitamin C; AECUL, acetone extract of *C. Longa*. Each bar represents mean ± standard error of mean at *P* < 0.05. ^*^Significant difference compared with group 1 (Control); ^µ^Significant difference compared with group 2 (CIM); ^β^Significant difference compared with group 3 (CIM + Vit. C); ^δ^Significant difference compared with group 4 (CIM + 200 mg/kg AECUL)

The plasma level of FSH (mIU/mL) was significantly lower in group 2 (0.18 ± 0.01) compared with group 1 (0.26 ± 0.01) (*P* < 0.05). No significant differences were recorded when the standard treatment group 3 (0.23 ± 0.01), AECUL‐treated groups 4, 5 and 6 (0.23 ± 0.02, 0.25 ± 0.02 and 0.24 ± 0.01, respectively), and group 7 (0.26 ± 0.01) were compared with group 1 (0.26 ± 0.01) (*P* > 0.05) (Figure 1A).

Group 2 (0.23 ± 0.02) had a significantly lower plasma LH level (mIU/mL) compared with group 1 (0.36 ± 0.01) (*P* < 0.05). However, no significant differences were recorded when the standard treatment group 3 (0.30 ± 0.02), AECUL‐treated groups 5 and 6 (0.34 ± 0.02 and 0.33 ± 0.01, respectively) and group 7 (0.37 ± 0.02) were compared with group 1 (0.36 ± 0.01) (*P* > 0.05) (Figure 1B).

The plasma testosterone level (ng/mL) was significantly lower in group 2 (0.25 ± 0.01) compared with group 1 (0.37 ± 0.01) (*P* < 0.05). No significant differences were recorded when the standard treatment group 3 (0.32 ± 0.02), AECUL‐treated groups 4, 5 and 6 (0.33 ± 0.02, 0.34 ± 0.02 and 0.33 ± 0.02, respectively) and group 7 (0.38 ± 0.01) were compared with group 1 (0.37 ± 0.01) (*P* > 0.05) (Figure 1C).

### 3.4 Effects of AECUL on oxidative stress and lipid peroxidation indicators in the brain and testis of Wistar rats with cimetidine‐induced pituitary‐testicular dysfunction

**Figure 2:**
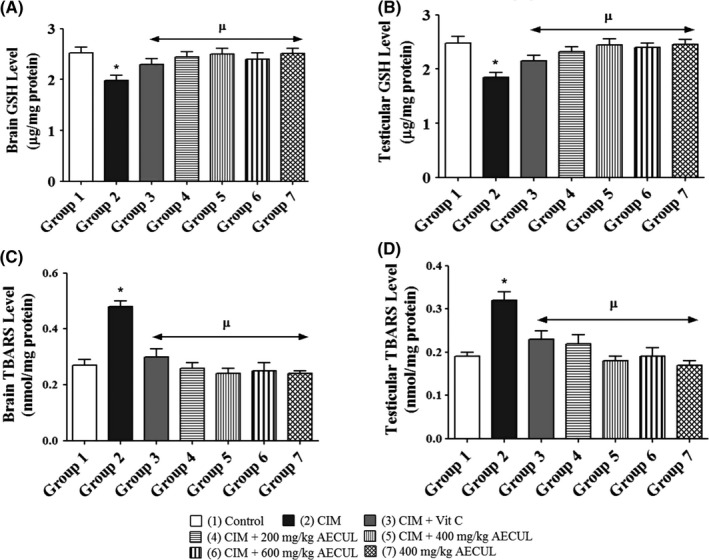
Effects of acetone extract of *Curcuma longa* on brain and testicular GSH and TBARS levels in Wistar rats with cimetidine‐induced pituitary‐testicular injury. GSH, reduced glutathione; TBARS, thiobarbituric acid reactive substance; CIM, cimetidine; Vit C, vitamin C; AECUL, acetone extract of *C. Longa*. Each bar represents mean ± standard error of mean at *P* < 0.05. ^*^Significant difference compared with group 1 (Control); ^µ^Significant difference compared with group 2 (CIM)

The brain GSH level (µg/mg protein) was significantly lower in group 2 (1.98 ± 0.10) compared with group 1 (2.52 ± 0.12) (*P* < 0.05). However, no significant differences were recorded when groups 3, 4, 5, 6 and 7 (2.30 ± 0.11, 2.45 ± 0.10, 2.50 ± 0.11, 2.40 ± 0.12 and 2.51 ± 0.11, respectively) were compared with group 1 (2.52 ± 0.12) (*P* > 0.05) (Figure 2A).

The testicular GSH level (µg/mg protein) was significantly lower in group 2 (1.85 ± 0.09) compared with group 1 (2.48 ± 0.12) (*P* < 0.05). However, no significant differences were recorded when groups 3, 4, 5, 6 and 7 (2.15 ± 0.10, 2.32 ± 0.09, 2.45 ± 0.11, 2.40 ± 0.08 and 2.46 ± 0.09) were compared with group 1 (2.48 ± 0.12) (*P* > 0.05) (Figure 2B).

The brain TBARS level (nmol/mg protein) was significantly lower in group 2 (0.48 ± 0.02) compared with group 1 (0.27 ± 0.02) (*P* < 0.05). No significant differences were recorded when groups 3, 4, 5, 6 and 7 (0.30 ± 0.03; 0.26 ± 0.02; 0.24 ± 0.02; 0.25 ± 0.03 and 0.24 ± 0.01, respectively) were compared with group 1 (0.27 ± 0.02) (*P* > 0.05) (Figure 2C).

The testicular TBARS level (nmol/mg protein) was significantly lower in group 2 (0.32 ± 0.02) compared with group 1 (0.19 ± 0.01) (*P* < 0.05). No significant differences were recorded when groups 3, 4, 5, 6 and 7 (0.23 ± 0.02, 0.22 ± 0.02, 0.18 ± 0.01, 0.19 ± 0.02 and 0.17 ± 0.01 respectively) were compared with group 1 (0.19 ± 0.01) (*P* > 0.05) (Figure 2D).

### 3.5 Histological effects of AECUL on the pituitary and testis of Wistar rats with cimetidine‐induced pituitary‐testicular dysfunction

**Figure 3:**
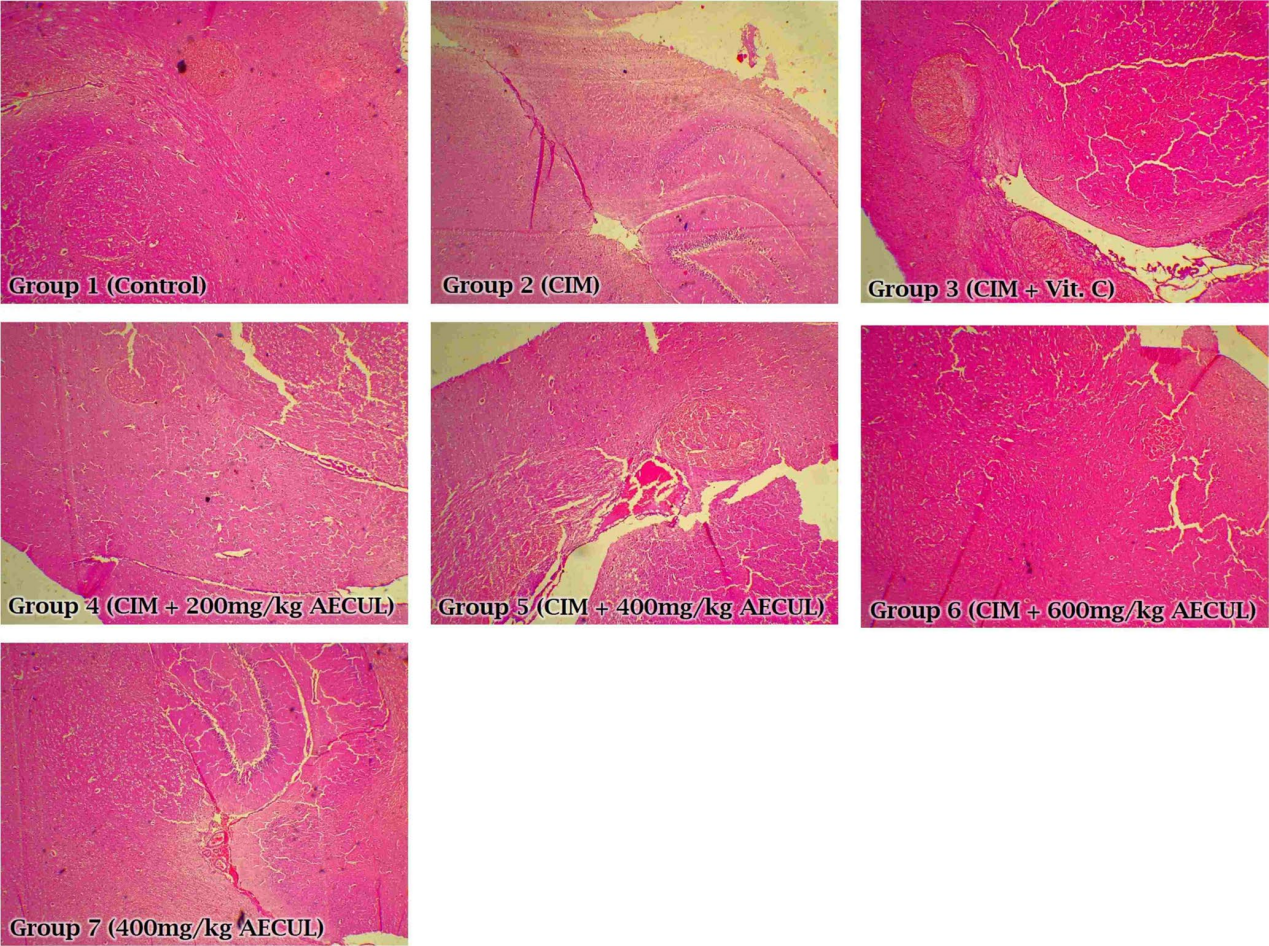
Histological effects of acetone extract of *Curcuma longa* on the pituitary of Wistar rats with cimetidine‐induced pituitary‐testicular injury. Magnification, ×40; CIM, cimetidine; Vit C., vitamin C; AECUL, acetone extract of *C. Longa*

Cimetidine administration (group 2) was associated with histoarchitectural distortion of the pituitary interstitium, with micrographic evidence of sparsely distributed pituitary cells compared with the control and the AECUL‐treated groups. The micrographic evidence from group 7 (treated with AECUL alone) showed features of apparently intact pituitary histoarchitecture compared with the control (Figure 3).

**Figure 4:**
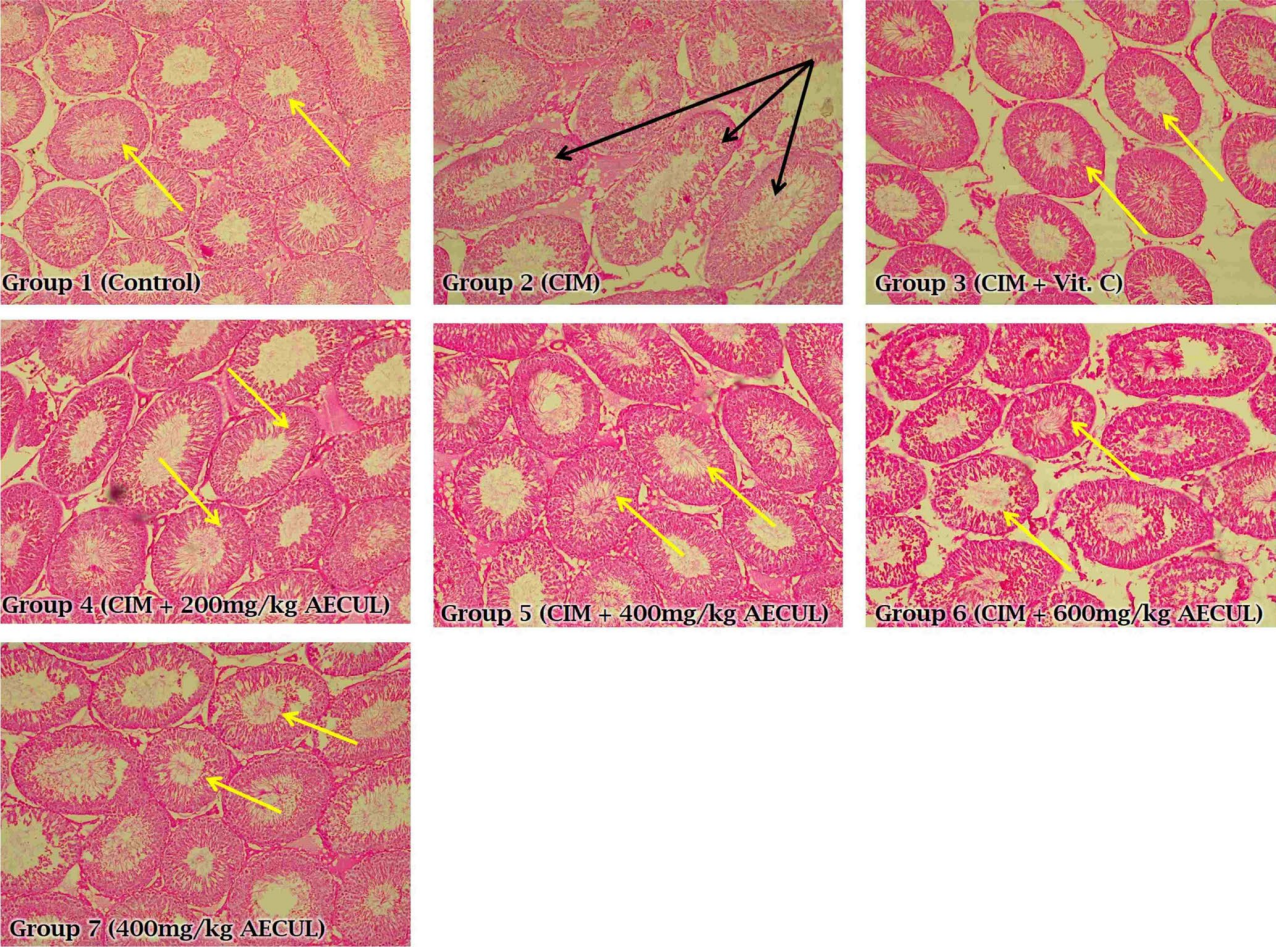
Histological effects of acetone extract of *Curcuma longa* on the testis of Wistar rats with cimetidine‐induced pituitary‐testicular injury. Magnification, ×100; CIM, cimetidine; Vit C., vitamin C; AECUL, acetone extract of *C. Longa*. Black arrow, ballooned and abnormally shaped/elongated seminiferous tubule with evidence of mild vacuolation of testicular interstitium; Yellow arrow, apparently intact seminiferous tubule

The micrographic evidence also showed features of cimetidine‐associated distortion of testicular histoarchitecture, characterized by ballooned/abnormally shaped seminiferous tubules and mild vacuolation of the testicular interstitium compared with micrographs from the control and AECUL‐treated groups (Figure 4).

## 4 DISCUSSION

Our study demonstrated that cimetidine‐induced reproductive toxicity is not accompanied by disturbances in body weight or relative brain and testicular weights of Wistar rats. This supports the findings of Qamar et al,^55^ who reported that cimetidine had no effect on testicular and body weights of Wistar rats. However, in our study, cimetidine induced deleterious alterations in the reproductive function of Wistar rats without causing significant changes in their relative testicular weight.

Cimetidine is involved in the control of multiple hormone secretory patterns by blocking the receptors for these hormones, thereby altering hormone profiles.^56^, ^57^ Furthermore, it has been reported that cimetidine penetrates the blood‐brain barrier^58^ to inhibit the synthesis of reproductive hormones.^59^ The micrographic evidence in our study showing scanty parenchymal cells in the cimetidine‐treated (toxic) group supports these reports and indicates an injurious effect of cimetidine on the pituitary, with consequent effects on pituitary secretions. The reduced plasma FSH level that accompanied cimetidine administration can be attributed to its possible deleterious effect on pituitary function and/or a possible decreased response of sertoli cells to circulating FSH. As a result of reduced LH following cimetidine‐induced reproductive toxicity, the recorded testosterone level decreased, which invariably caused impairment of spermatogonia proliferation by reducing its active binding to the available FSH. The increased level of these reproductive hormones accompanying AECUL administration demonstrates the fertility‐boosting potential of the extract. Some phytochemicals in the extract are flavonoids and tannins which are reputed to have antioxidant and anti‐inflammatory potential.^60^, ^61^ Since cimetidine caused alterations in the antioxidant system, AECUL may have reversed the suppression of hormonal release and expression via restoration of normal antioxidant status.

Sperm count is one of the most reliable and sensitive tests for spermatogenesis; it has a high correlation with fertility because it provides information on the cumulative result of all the stages of sperm production.^62^, ^63^, ^64^ The cimetidine‐induced decrease in sperm count demonstrates impairment of reproductive function via injurious interference with the critical stages of spermatogenesis. This suggested that AECUL boosted sperm counts by providing protection against the effects of cimetidine. Sperm motility and viability are also important and integral parameters of many reproductive toxicity guidelines.^10^ These sperm characterization indices can be affected when a chemical agent penetrates the blood‐testis barrier to cause deleterious changes in the micro‐environment of the seminiferous tubules.^65^ This may explain the abnormally shaped seminiferous tubules seen in the group treated with cimetidine alone. It is possible that AECUL possesses some phytochemicals that penetrate the blood‐testis barrier and restore the micro‐environment of the seminiferous tubules to re‐establish normal spermatogenesis. If further evidence of this effect is forthcoming, it would be worth identifying the active phytochemicals in AECUL to establish their pharmacological activities.

Reduced glutathione (GSH) is a non‐enzymatic antioxidant marker, while thiobarbituric acid reactive substance (TBARS) is an important index of lipid peroxidation.^10^, ^42^ This study demonstrated that cimetidine administration caused deleterious disturbance of the antioxidant system. We suggest that the increased use of GSH to scavenge the free radicals generated by cimetidine resulted in significantly reduced GSH levels in the group treated with cimetidine alone. The restoration of antioxidant levels by AECUL suggests that the extract has potent antioxidant activity, as well as the potential to reduce cellular disruption by chemical agents, as demonstrated by the comparison of TBARS levels. Some of the phytochemicals identified in AECUL, such as flavonoids, phenolics and saponins, have been reported in the literature to have potent antioxidant, anti‐inflammatory and membrane‐stabilizing potential.^41^, ^42^ Therefore, we conclude that the overall pharmacological (including antioxidant) activity of the extract was conferred by the presence of these important phytochemicals.

Generally, the extract demonstrated better therapeutic potential as well as better preservation of pituitary and testicular histoarchitecture when compared with the standard treatment group (co‐administration of vitamin C). This suggests that the therapeutic dose of vitamin C (50 mg/kg) 66, 67 adopted for research purposes may not be adequate in the management of subjects with cimetidine‐induced reproductive toxicity. As a result of our study, we recommend either a review of this therapeutic dose or that it is used as an adjunct to other interventions in the management or treatment of cimetidine‐induced reproductive toxicity. In addition, the study did not demonstrate any dose‐dependent response of the extract. This suggests that lower doses of the extract (200 and 400 mg/kg) may be adopted for further assessments of the therapeutic potentials of AECUL, since the highest dose (600 mg/kg) showed an apparent (but not significant) decline in most of the assessed parameters compared with the medium dose (400 mg/kg). Notably, administration of the extract alone did not cause any apparent deleterious biochemical and histological alterations, indicating that the extract alone does not alter normal reproductive function in Wistar rats. A study of the prophylactic effects of AECUL in cimetidine‐induced reproductive toxicity is highly recommended.

This study concludes that an acetone extract of *C. Longa* normalized cimetidine‐induced pituitary‐testicular dysfunction in Wistar rats, apparently via potent antioxidant and membrane‐stabilizing mechanisms. This presents the extract as a potential nutraceutical choice against chemically induced reproductive toxicity.

## CONFLICT OF INTEREST

None.

## AUTHOR CONTRIBUTIONS

AAO initiated the research idea and supervised the study. ONJ and ICE conceptualized the study. NJO was responsible for fund acquisition. ICE and ONJ performed the data analyses. All authors participated in the acquisition and interpretation of data. All authors were involved in drafting, revising and proof‐reading the article for intellectual content.
